# Tissue distribution and differential expression of melanocortin 1 receptor, a malignant melanoma marker

**DOI:** 10.1038/sj.bjc.6600441

**Published:** 2002-08-12

**Authors:** F Salazar-Onfray, M López, A Lundqvist, A Aguirre, A Escobar, A Serrano, C Korenblit, M Petersson, V Chhajlani, O Larsson, R Kiessling

**Affiliations:** Disciplinary Program of Immunology, Institute of Biomedical Sciences, Faculty of Medicine, University of Chile, Av. Independencia 1027, Santiago, Chile; Department of Oncology and Pathology, Cancer Center Karolinska (CCK), S-171 76 Stockholm, Sweden; Lead Discovery Department AstraZeneca, Wilmington, Delaware, DE 19850, USA

**Keywords:** melanocortin 1 receptor, melanoma antigen, dendritic cells, tumour marker, tumour immunology

## Abstract

The melanocortin 1 receptor is a G-protein-coupled receptor, described to be expressed on melanomas and melanocytes. Subsequent RT–PCR studies demonstrated the presence of melanocortin 1 receptor mRNA in other tissues such as pituitary gland and testis. Previously, we have demonstrated that three HLA-A2 binding nonamer peptides derived from melanocortin 1 receptor can elicit peptide-specific CTL which can recognize target cells transfected with the melanocortin 1 receptor gene and MHC class I matched melanoma lines. The potential of targeting melanocortin 1 receptor in therapy and diagnosis will depend on a preferential expression of this receptor in the majority of primary and metastatic melanomas *vs* normal tissues. We tested a panel of melanomas, carcinomas and other cell lines for the presence of melanocortin 1 receptor, using two monoclonal antibodies. The receptor was detected in 83% of the tested melanoma cell lines but not in other carcinoma lines. Immunohistochemistry revealed a strong expression of melanocortin 1 receptor in all tested primary and metastatic melanomas, but also demonstrated low levels of expression in adrenal medulla, cerebellum, liver and keratinocytes. Flow cytometry studies showed that melanocortin 1 receptor was expressed in *in vitro* activated monocytes/macrophages and in the THP-1 monocytic leukaemia line at levels of about 1 in 3 to 1 in 5 of that found in melanomas. Peripheral blood-derived dendritic cells, also express melanocortin 1 receptor *in vitro*. This extensive analysis of melanocortin 1 receptor tissue distribution may be of relevance not only for melanoma immunology, but also for research on the pathogenicity of inflammatory conditions in the skin and neurologic tissues. It remains to be seen if the over-expression of melanocortin 1 receptor in melanomas is sufficiently high to allow a ‘therapeutic window’ to be exploited in cancer immunotherapy.

*British Journal of Cancer* (2002) **87**, 414–422. doi:10.1038/sj.bjc.6600441 www.bjcancer.com

© 2002 Cancer Research UK

## 

Alpha-melanocyte stimulating hormone (α-MSH) is a tridecapeptide derived from the precursor molecule propiomelanocortin (POMC) (Eberle, 1988) and is primarily released by the pituitary, but also by immunocompetent cells (Schauer *et al*, 1994; Farooqui *et al*, 1995; Star *et al*, 1995). α-MSH is a very potent stimulator of the pigmentation and differentiation of pigmented cells, including melanoma cells (Schwahn *et al*, 2001). POMC derived peptides have been shown to transmit their signal via G-protein coupled receptors through the formation of cyclic AMP and activation of protein kinase C (Friedmann *et al*, 1990; Ao *et al*, 1998; Abdel-Malek *et al*, 2000). Five different subtypes of melanocortin receptors with different tissue distribution have been described (Chhajlani and Wikberg, 1992; Mountjoy *et al*, 1992; Chhajlani *et al*, 1993; Gantz *et al*, 1993a,b). Of these, the melanocortin 1 receptor (MC1R) was originally described to be mainly located on melanoma and melanocytes (Varga *et al*, 1976; Tatro *et al*, 1990; Chhajlani, 1996; Suzuki *et al*, 1996; Xia *et al*, 1996). MC1R was, however, subsequently shown also to be expressed in various human tissues including human keratinocytes and monocytes (Chhajlani ans Wikberg, 1992; Rajora *et al*, 1996; Thörnwall *et al*, 1997; Chakraborty *et al*, 1999; Curry *et al*, 2001).

The cloning of genes encoding human melanoma-associated antigens, and the definition of CTL peptide epitopes derived from these, has stirred considerable interest among immunologists and clinicians. The majority of the characterized melanoma-associated CTL epitopes are derived from normal, non-mutated proteins. These include the melanin synthesis associated proteins Melan-A/Mart-1, gp100, and tyrosinase (Brichard *et al*, 1993; Coulie *et al*, 1994; Kawakami *et al*, 1994a,b), which encode peptide CTL epitopes presented by MHC Class I molecules (Brichard *et al*, 1993; Kawakami *et al*, 1994c; Kawakami *et al*, 1995). These antigens are also expressed on normal melanocytes in the skin and on melanocyte-like cells in the retina, and therefore should be regarded as true ‘self’ antigens. CTL reacting with these antigens can often be demonstrated in patients with advanced melanomas (Brichard *et al*, 1993; Coulie *et al*, 1994; Kawakami *et al*, 1994a,b,c, 1995). These results have been interpreted as evidence for the breaking of immunological tolerance to normal cellular proteins, perhaps due to overexpression of these molecules on tumour cells.

Recently, we have demonstrated that three MC1R derived nonameric peptides which bind with high- or intermediate affinity to HLA-A2 can generate peptide specific CTL from peripheral blood mononuclear cells (PBMC) of healthy HLA-A2+ donors. These peptide-specific CTL could also recognise HLA-A2+ melanoma cells expressing MC1R, demonstrating that the MC1R derived peptides are naturally processed and presented by MHC class I on the surface of melanoma cells (Salazar-Onfray *et al*, 1997).

The possibility of using MC1R for antibody or T cell based immunotherapy is dependent on how selectively this receptor is expressed in melanomas *vs* normal tissues. Although there is evidence that MC1R has a broad distribution in various normal tissues including normal melanocytes, activated macrophages, and keratinocytes (Chhajlani, 1996; Rajora *et al*, 1996; Thörnwall *et al*, 1997; Chakraborty *et al*, 1999; Curry *et al*, 2001), this assessment is based mainly on qualitative or semi-quantitative methods. There is experimental and clinical evidence that immunotherapy based on administration of monoclonal antibodies or on specific tumour vaccines can also have an effect when based on non-mutated ‘self-proteins’ over-expressed in tumours, such as non-mutated p53, HER-2/neu, and gp100 (Ropke *et al*, 1996; Nagata *et al*, 1997; Rosenberg *et al*, 1998; Buchler *et al*, 2001; Slamon *et al*, 2001). This requires however that the level of over-expression in the tumour, as compared to normal tissue, is high enough to allow a ‘therapeutic window’, allowing tumour eradication without excessive autoimmune damage.

In the present study we have analysed several questions relevant to the potential use of MC1R in immunotherapy of melanomas. Using flow cytometry based methodology to measure intracellular levels of MC1R, Western blot and immunohistochemistry, we verify that this molecule is expressed at high levels in the majority of melanoma lines and in fresh primary and metastatic melanomas, and at low levels in certain normal tissues. Of particular interest, *in vitro* activated monocytes and dendritic cells (DC) produced *in vitro*, also express MC1R although at lower levels than that observed in melanomas.

## MATERIALS AND METHODS

### Reagents and chemicals

Biotinylated-α-melanocyte stimulating hormone (Biotinyl-α-MSH) was purchased from Peninsula laboratory (CA, USA) and non-labelled α-MSH was purchased from Sigma (Steinheim, Germany). Phycoerythrin (PE)-conjugated streptavidin was purchased from Pharmingen (San Diego, USA).

### Cell lines

All the melanoma cell lines were established at the Microbiology and Tumor Biology Center (MTC), Karolinska Institute. The lines were derived from metastatic lesions of patients treated at Radiumhemmet, Karolinska Hospital, except the lines 397mel kindly provided by Dr Y Kawakami NCI, Maryland, USA and FM3Dmel and FM55.M1mel kindly provided by Dr J Zeuthen, Cancer Society, Copenhagen, Denmark. DFW is a depigmented melanoma subline obtained from DFB by limiting dilution. OCM1 and OCM3 are ocular melanoma lines kindly provided by Dr M Jager (University of Leiden). OCM5 is an uveal melanoma established at the Institute of Biomedical Sciences. The T2 line is a TAP-defect cell line derived from the human T cell leukemia/B cell LCL hybrid 174 (Salter and Cresswell, 1986). C1R-A2 is a class I defective cell line transfected with HLA-A2 (Zemmour *et al*, 1992) and C1R-A2/MC1R is the same line transfected with the MC1R gene. SW480 and SW620 are colon cancer cells from American Type Culture Collection (ATCC). OVA3507, OVA6906, are colon cancer cells from American Type Culture Collection (ATCC). OVA3507, OVA6906, and AK12, are ovarian carcinoma cell lines established at the MTC. CAOV-4 is an ovarian carcinoma from ATCC. THP-1 is a monocytic tumour cell line. Cell line 293 is an embryonal kidney tumour. BE EBV; BL EBV; 1224 EBV, and 0351 EBV are LCL lines established at the MTC. The human melanocytes cell cultures (kindly provided by Dr Luz Maria Muñoz, Faculty of Medicine, University of Chile) were maintained in Melanocyte Basal Medium (Sigma, St. Louis, MO, USA).

### *In vitro* culture of monocytes and dendritic cells

Cells from human buffy coats were isolated by separation with Ficoll-Hypaque (Pharmacia, Sweden). All cells were incubated in RPMI medium and 10% FCS for 2 h (20×10^6^ cells ml^−1^) in a 6 well plate (Costar, Cambridge, MA, USA). Then, non-adherent cells were removed and the adherent cells were recovered and prepared for Western blot (fresh monocytes) or left with only medium (non stimulated monocytes) or stimulated for 48 h with LPS (2.5 μg ml^−1^; Sigma Chemical Co., Munich, Germany), PHA 2 μg ml^−1^, human recombinant IL-4 (rIL-4) 100 U ml^−1^ or GM-CSF 100 U ml^−1^ (generously donated by Shering Plough, Brinny Co., Ireland). Dendritic cells were isolated in the same manner as monocytes and incubated with 100 U ml^−1^ of GM-CSF and 100 U ml^−1^ of rIL-4 (also donated from Shering Plough) for at least 7 days. The medium was replaced every 3–4 days and the cultures were maintained for 7–9 days. PE-conjugated anti-CD14 (Beckton-Dickinson, Mountain View, CA, USA) and anti-CD36 (BD PharMingen, San Diego, CA, USA) were used for the analysis of cell surface phenotype of monocytes and dendritic cells respectively. FITC-conjugated CD83 (BD PharMingen, San Diego, CA, USA) were used to determine the maturation of the DC.

### Western blot

Cell pellets from harvested melanoma cell lines (1*10^6^ cells) were suspended in 100 μl lysis buffer (65 mM Tris pH 6.8, 2% SDS, 10% glycerol, 5% mercapto-ethanol, 1% bromophenol blue) and maintained for 15 min at room temperature. Then, samples were sonicated by 10 microns for 30 s, warmed at 95°C for 5 min and then centrifuged. Fifteen μl each sample was electrophoresed through a 12% SDS-polyacrylamide gel. For immunoblots, proteins were electro transferred onto polyvinyldene fluoride (PVDF) membrane (Immobilon-P; Millipore Corp., Bedford, MA, USA). Membranes were blocked in phosphate-buffered saline containing 5% milk (low fat) (PBS/milk 5%). All additional immunostaining steps were performed in phosphate-buffered saline with 3% milk (PBS/milk 3%) and washed with phosphate-buffered saline 0.05% Tween20 (PBS-Tween 0.05%) at room temperature. Filters were incubated overnight at 4°C with the corresponding primary antibody; (MP1-1B7 or MP1-1C11) (Thörnvall *et al*, 1997), for 1 h and then with a secondary antibody (horseradish peroxidase-conjugated sheep anti-mouse Ig; Amersham, Buckinghamshire, UK) for 30 min. As an internal control a commercial polyclonal antibody against β-actin was used. Filters were then washed in PBS-Tween four times and developed with enhanced chemiluminescence (ECL) system (Amersham, Buckinghamshire, UK).

### MC1 receptor binding studies

All procedures were performed according to previously described methods (Chhajlani *et al*, 1993). Briefly, 2×10^6^ cells were collected and washed with PBS, acidic glycine buffer and RPMI medium. After transfer of 2×10^5^ cells per well in a 96-well plate, 10^−10^ M biotin-labelled α-MSH were added to each well and the cells were incubated for 1 h at 37°C. Then the cells were washed with cold PBS once and incubated with PE-labelled streptavidin (40 μg ml^−1^) for 30 min at 4°C in the dark. As control, cells were incubated in the presence of PE-streptavidin only. The specificity of the biotin-labelled α-MSH binding was evaluated by the addition of unlabelled α-MSH. The cells were analysed using a FACScan flow cytometer (Becton and Dickinson) after fixation with 1% formaldehyde in PBS.

### Intracellular staining

Analysed cells were washed three times with cold PBS, fixed by 10 min incubation with 0.5% paraformaldehyde in PBS, and washed again with PBS. The cells were permeabilized with 2% digitonin (Sigma, St. Louis, MO, USA) in PBS for 10 min on ice. After checking the permeability of the cells by trypan blue uptake, 3×10^5^ cells per well were added to a 96 v-bottom well and incubated with 5 μg ml^−1^ of mAb MP4-1B7 (anti MC1R) (Thörnvall *et al*, 1997), 2 μg ml^−1^ of mAb 9.2.27 (Morgan *et al*, 1981) or control IgG1 mAb for 30 min on ice. Cells were washed twice with PBS and incubated 30 min on ice, with a secondary rabbit anti-mouse Ig PE-conjugated antibody (DAKO, Denmark). After incubation, the cells were washed three times with PBS 0.01% Tween. Then the cells were fixed again with 1% paraformaldehyde in PBS containing 0.1% FCS and kept at 4°C until analysis by FACS.

### Immunohistochemical analysis

Normal and some melanoma tissues were obtained from the Department of Oncology and Pathology, Karolinska Hospital. The majority of primary and metastatic melanoma tissues were obtained from the Department of Pathological Anatomy of the Hospital of University of Chile. Tissues were fixed in paraformaldehyde, embedded in paraffin, and sectioned. Immunostaining was performed using the standard ABC-technique (Dako LSAB kit, CA, USA). Paraffin sections were deparaffinized and rehydrated. The endogenous peroxidase activity was blocked by hydrogen peroxide (dissolved in methanol for 30 min). Sections were incubated with a blocking serum (normal horse serum) for 30 min. Excess of serum was drained and the sections were incubated with the primary antibody (MP1-1B7 or MP1-1C11 anti-MC1R) (Thörnvall *et al*, 1997) at a concentration of 10 μg ml^−1^. All incubations were performed overnight at 8°C. A biotinylated anti-mouse IgG was used as secondary antibody and followed by the ABC-complex. The peroxidase reaction was developed using 3,3-diaminobenzidine (diamino-benzidine tetrahydrochloride, 0.6 mg ml^−1^ with 0.03% hydrogen peroxide) for 6 min. Counter staining was not performed in a majority of cases. Haematoxylin solution (Fluka, Buchs, Switzerland) were used for nuclear staining in specified cases. Phosphate buffered saline (pH 7.6) was used for rinsing between the different steps. The MC1R protein expression was evaluated according to an arbitrary scale: 1,+,++,+++ on the basis of the intensity of the immunoreactivity.

### Characterization of melanomas

The cells were tested by DAKO EPOS visualisation system (Dakopatts AB, Älvsjö, Sweden). Briefly, cells were washed with PBS and then 2×10^5^ cells per well were fixed with methanol in a 96 v-bottom well. The cells were then washed with PBS and 50 ml of horseradish peroxidase (HRP)-coupled mAb HMB45 (Kapur *et al*, 1992), S-100 (Cochran *et al*, 1983) or IgG as control was added (Dakopatts). After 30 min incubation on ice, the cells were washed with PBS and 50 ml of the substrate (DAB) (Dakopatts) was added. Then, the cells were checked in the microscope for colour reaction.

## RESULTS

### MC1R is frequently expressed in melanoma cell lines

**Figure 1:**
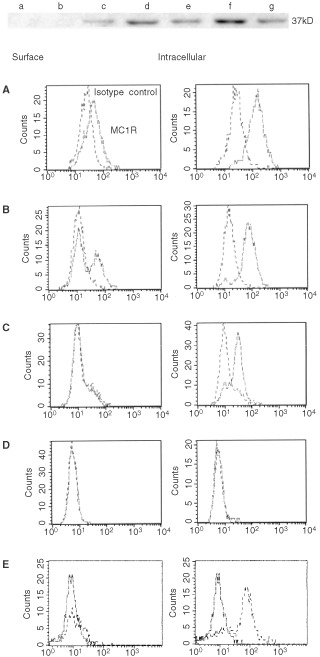
Expression of MC1R in melanoma cell lines. Upper panel: Cell lines (a) C1R-A2, (b) C1R-A2 neo, (c) C1R-A2/MC1R(1), (d) C1R-A2/MC1R(2), (e) FMS, (f) OCM1, (g) OCM3 were analysed by Western blot for the presence of the 37 kD band corresponding to MC1R. Lower panel: DFB melanoma. (**A**), FM55 melanoma (**B**), BL melanoma (**C**), C1R-A2 LCL line as a negative control (**D**), and C1R-A2/MC1R (**E**) were fixed and incubated with mAb MP4-1B7 directly (left) or after permeabilisation with digitonin (right) and then stained with PE-conjugate anti-IgG mAbs and analysed by FACS as described in Materials and Methods. The experiments were performed twice with similar results.

The specificity of the mAbs MP1-1B7 and MP1-1C11, previously described to be specific for the extracellular domain of MC1R (Thörnvall *et al*, 1997), was confirmed by Western blot analysis on melanoma lines and EBV immortalized B cell lines (LCL). Three melanoma cell lines (FMS, OCM3 and OCM1) were positive for a 37 kD protein, which corresponds to the expected size of MC1R, while the LCL line C1R-A2 was negative. C1R-A2 transiently transfected with the vector pRC/CMV-hMCIR was positive for MC1R, confirming the specificity of the antibody (Figure 1

, upper panel, shown for mAb MP1-1C11, similar results were observed using the mAb MP1-1B7, data not shown). Western blot analysis using cryo-preserved melanoma tissues derived from a metastatic lymph node also demonstrated a strong band corresponding to MC1R in both analysed melanomas, with similar intensity of expression as that observed in the *in vitro* cultured melanoma lines (data not shown).

Intracellular *vs* cell surface expression of MC1R was also analysed by flow cytometry on permeabilized and non-permeabilized melanoma and LCL lines. Both the DFB and FM55 melanoma lines showed a weak but significant surface expression of MC1R (Figure 1A,B) while the BL melanoma line and the C1R-A2 line were completely negative (Figure 1C,D). Permeabilized cells showed considerably stronger signalling than surface stained ones, and all three tested melanomas, including the cell surface negative BL line, stained positive with the anti MC1R mAb (Figure 1A–C), while the C1R-A2 line remained negative (Figure 1D). The MC1R transfected cell line, C1R-A2/MC1R showed a major population of MC1R-positive cells, although a minor population remained negative, probably due to low transfection efficiency and/or incomplete selection (Figure 1E). We therefore conclude that melanoma lines which do not stain for cell surface expression can still show intracellular expression of this protein, the reason why this method of intracellular staining was used in the subsequent screening of MC1R expression in tumour lines.

We next performed a more extensive analysis of the intracellular expression of MC1R in a series of melanomas and carcinomas. The majority of the melanoma cell lines (20 out of 24), including three ocular melanomas were found to express this receptor (Table 1

**Table 1:**
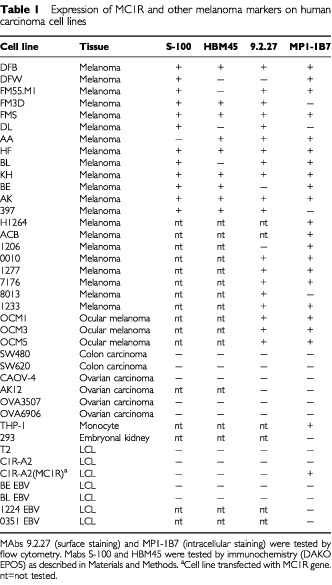
Expression of MC1R and other melanoma markers on human carcinoma cell lines

). The MC1R transfected C1R-A2 was as expected strongly positive for MC1R, while the control C1R-A2 line and five other LCL were negative. In addition, two colon carcinomas, four ovarian carcinomas, and one embryonal kidney line did not express MC1R.

Then, we compared the expression of MC1R with that of other commonly expressed melanoma markers. Among the melanoma lines which were positive for the S-100 mAb (12 out of 13), three did not express MC1R (FM3D, DL and 397). Conversely, among the 20 melanomas which expressed MC1R, one (AA) was not positive for the S-100 marker. None of the tested carcinomas or LCL was, as expected, positive for the S-100 marker.

Among the melanomas which were positive for the HBM45 mAb (8 out of 13), two did not express MC1R (FM3D and 397). Conversely, two of the HBM45 negative melanomas expressed MC1R, including the depigmented variant DFW of the DFB line and the BL line, while one lacked the expression of both these markers (DL) while still staining positive for the S-100 mAb. None of the tested carcinomas or LCL was, as expected, positive for the HBM45 marker.

We also tested the surface expression of the protein recognised by the melanoma specific mAb 9.2.27 (Morgan *et al*, 1981). This antibody recognises the melanoma-associated chondroitin sulfate proteoglycan (MCSP) (Pluschke *et al*, 1996) expressed on melanomas (Morgan *et al*, 1981) and astroglial malignant cells (Schrappe *et al*, 1991), and is suggested to play a role during early events of melanoma cell spreading (Morgan *et al*, 1981). Only three of the 22 melanomas (DFW, BE and 1206) were negative for this mAb, all three of which expressed detectable levels of MC1R. Four melanomas that were recognised by mAb 9.2.27 were negative for MC1R (FM3D, DL, 397 and 8013). All carcinomas and LCL were, as expected, negative for the 9.2.27 marker.

We therefore concluded that MC1R is expressed in the majority of melanoma cell lines but in none of the tested carcinomas or LCL, and that the expression of this receptor does not correlate with any of the other tested melanoma markers.

### Immunohistochemical detection of MC1R on paraffin-embedded human tissues

**Figure 2:**
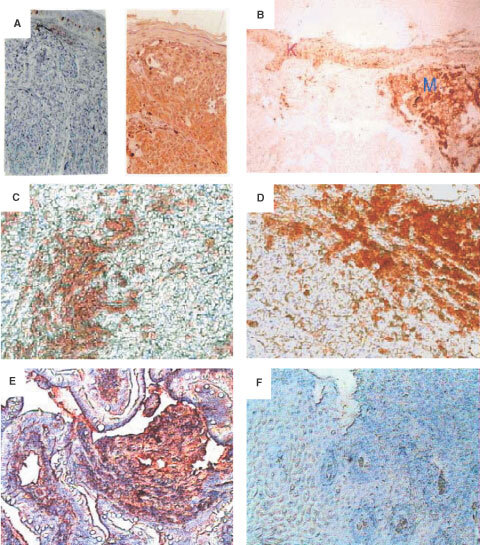
Immunohistochemical detection of MC1R on primary melanoma tissues, normal keratinocytes and metastatic melanoma tissues. Paraffin sections of primary melanoma tissues and normal skin tissues and metastatic melanoma tissues were stained with mAb MP1-1B7 or IgG control followed by a biotinylated anti-Ig mAb and developed as described in Materials and Methods. (**A**) normal naevus 10× (left) and primary melanoma (right) (**B**) Comparison of the intensity of labelling between a primary melanoma (M) and keratinocytes (K)×10. (**C**) Melanoma infiltrated lymph node (MP1-1B7)×10. (**D**) Maxilar metastasis (MP1-1B7)×10. (**E**) Intestinal metastatic melanoma (MP1-1B7)×10, and (**F**) Intestinal metastatic melanoma (Ig negative control)×10. In panels **A**, **E**, and **F**, nuclear staining was performed using haematoxylin solution.

Paraffin sections from melanomas or normal tissues were analysed by immunohistochemistry for MC1R expression. An intense specific staining was detected in all tested primary melanomas (*n*=15) (exemplified in Figure 2A

(right panel) and 2B (M)), including three ocular melanomas. Also, all metastatic melanomas (*n*=11) stained strongly positive for MC1R, including metastasis in lymph nodes (Figure 2C), maxilla (Figure 2D), intestine (Figure 2E), while the adjacent normal tissues, including the epidermis, dermis, appendages, and subcutaneous tissue and connective tissues, were negative. A metastatic melanoma from intestine stained with an irrelevant Ig was used as negative control (Figure 2F). Melanocytes from normal naevi were also negative for MC1R expression (Figure 2A (left panel)). It was previously described that human keratinocytes could specifically bind to α-MSH (Chakraborty *et al*, 1999; Curry *et al*, 2001). We confirmed the presence of MC1R in keratinocytes (Figure 2B (K)), although the intensity of staining was weaker than that observed in the melanoma tissue from the same skin. No differences were detected in the intensity of staining between primary and metastatic melanoma tissues.

Using the same method, MC1R was also detected in some cell components of normal tissues, including adrenal medulla, cerebellum, and liver, although with a considerably lower intensity as compared to the melanomas (Table 2Table 2

**Table 2:**
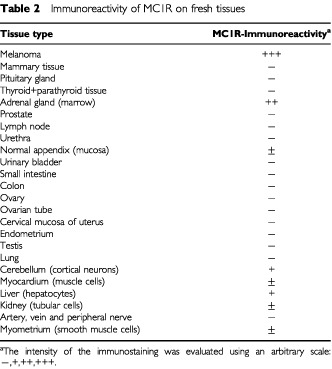
Immunoreactivity of MC1R on fresh tissues

). A very weak staining, at the border of being significant, was observed in normal appendix, myocardium, kidney, and myometrium. No specific staining was observed in the other 16 analysed normal tissues (Table 2). Since melanocytes were not stained by MC1R either *in situ* in the melanoma surrounding tissue or in normal naevi, we intend to detect MC1R expression in short time cultured melanocytes. To perform this, melanocytes which had been cultured for two passages *in vitro* were analysed by immunocytochemistry as described in Materials and Methods. Both melanocyte tissues analysed here were found to express MC1R (Figure 3A,B

**Figure 3:**
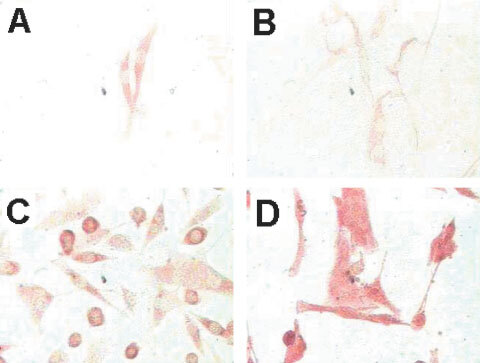
MC1R is overexpressed on melanomas and low expressed in *in vitro* cultured melanocytes. Melanoma, normal melanocytes cells and control COS7 cells were cultured on an objective glass overnight, carefully washed with PBS and then fixed with methanol. The fixed cells were stained with mAb MP1-1B7 or IgG control followed by a biotinylated anti-Ig mAb and developed as described in Materials and Methods. (**A** and **B**) Normal melanocytes, (**C**) FM55 melanoma line, and (**D**) FMS melanoma line.

), although the intensity of the staining was significantly lower than that observed in two melanoma cell lines FM55 (Figure 3C) and FMS (Figure 3D), tested in parallel. Collectively, these results demonstrate that MC1R, although strongly expressed in melanomas, is also expressed at lower levels in several normal tissues *in situ*.

### MC1R can also be detected in human* in vitro* stimulated monocytes and in a monocytic leukaemia line

**Figure 4:**
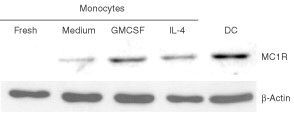
*In vitro* stimulated monocytes and immature DC cells express MC1R. Fresh monocytes, monocytes incubated with medium alone, IL-4 or GM-CSF, and *in vitro* produced DC were analysed as described in Materials and Methods by Western blot for the presence of the 37 kD band corresponding to MC1R. As external control, the samples were analysed for the expression of β-actin.

The induced expression of MC1R was previously demonstrated on activated monocytes, using biotin-labelled α-MSH and flow cytometry analysis (Bhardwaj *et al*, 1997). To confirm the presence of MC1R in monocytes, we performed Western blot analysis using the anti MC1R mAb. Also, dendritic cells (DC) described to express MC1R mRNA (Becher *et al*, 1999), produced by culture of monocytes in a combination of IL-4 and GM-CSF for 7 days (Sallusto and Lanzavecchia, 1994), were included in this experiment. We observed that fresh untreated monocytes did not express detectable levels of MC1R, while monocytes cultured in medium alone showed very low expression of this receptor. Furthermore, we confirmed that *in vitro* culturing of monocytes in IL-4 or GM-CSF could induce the expression of MC1R (Figure 4

). In addition, LPS and PHA treatment of monocytes could induce MC1R expression (data not shown). We also investigated the expression of MC1R in immature DC (CD14-, CD36+, CD83-) (Banchereau and Steimann, 1998; Sauter *et al*, 2000). We observed that cytokine induced DC also expressed comparable levels of MC1R as those observed in cytokine stimulated monocytes (Figure 4).

**Figure 5:**
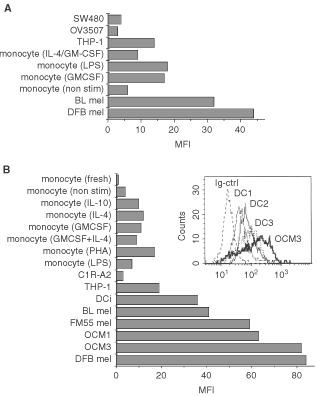
MC1R is overexpressed on melanomas compared to stimulated monocytes. (**A**) α-MSH binds specifically to melanoma cells and to stimulated monocytes. Cells were incubated with biotin-labelled α-MSH for 1 h, washed, stained with PE-conjugated streptavidin and then analysed by FACS as described in Materials and Methods. (**B**) Intracellular expression of MC1R in melanoma cell lines, the monocytic line THP-1 and stimulated monocytes. Five melanoma cell lines (DFB mel, FM55 mel and BL mel, OCM1 and OCM3), the macrophage derived line THP-1, 7 day cultured dendritic cells (DCi) (showed as mean of three lines in **B** and separately (DC1, DC2 and DC3 in the histogram) and nonstimulated monocytes or monocytes incubated with cytokines for 48 h were fixed, permeabilised and incubated with mAb MP1-1B7. PE-conjugated secondary mAb were used for detection by FACS as described in Materials and Methods. These experiments were performed three times each with similar results.

To further compare the levels of MC1R expression between melanomas and stimulated monocytes, we also performed flow cytometry analysis of melanomas and monocytes stained with fluorescein-conjugated α-MSH. This analysis showed that melanomas bind five to seven times more to α-MSH than monocytes cultured in medium alone (Figure 5A

). We also confirmed (Bhardwaj *et al*, 1997) that monocytes activated by short term culture with LPS or with GM-CSF alone or in combination with IL-4, showed enhanced binding of α-MSH (30% for IL-4/GM-CSF and 2–3-fold for GM-CSF or LPS treated monocytes) as compared to control monocytes cultured in medium only. The macrophage/monocyte line THP-1 showed the same levels of α-MSH binding as LPS- or cytokine stimulated monocytes (Figure 5A), confirming the MC1R expression in this line (Rajora *et al*, 1996), while an ovarian and a colon carcinoma (OV3507, SW480) included as controls only bound low to insignificant levels of α-MSH.

Next, we compared melanomas and cultured monocytes with regard to their intracellular content of MC1R, as measured by flow cytometry of permeabilised cells using the MP1-1B7 mAb. A more pronounced difference in intracellular expression of MC1R between melanoma lines and cultured monocytes was detected with this method as compared to that found with the α-MSH binding method. Thus, stimulated monocytes or the macrophage line THP-1 showed levels of intracellular MC1R expression which were 50–20% of that found in the five melanomas tested (MFI 8 to 18 for stimulated monocytes *vs* 40 to 85 for the melanomas) (Figure 5B). Fresh monocytes, monocytes cultured in only medium, and the negative control line C1R-A2 showed levels of expression similar to the non-specific control mAb (Figure 5B). No significant differences between monocytes or melanoma lines could be detected using an anti-Vimentine mAb as control (data not shown). Three different DC lines cultured for 7–9 days in medium containing GM-CSF and IL-4 (CD14^−^, CD36^+^ and CD83^−^) showed higher levels of intracellular MC1R than short time cultured monocytes, comparable to that observed in the low MC1R expressing BL melanoma cell line. These levels were however lower (30–50%) than those observed in the high MC1R expressing melanoma lines (DFB, FM55, OCM1 and OCM3) (Figure 5B).

Taken together, this confirms that MC1R is expressed at significant levels in activated monocytes, DC cells, and macrophage derived leukaemia cells, although to a lesser extent than in melanoma cell lines.

## DISCUSSION

The present report contains several new observations relevant to the potential use of MC1R in diagnostic and immunotherapy of melanomas. First, we demonstrate with flow cytometry the broad distribution of this protein in the majority of melanoma lines, and that the molecule predominantly is located intracellularly. Second, we utilise immunohistochemistry and Western blot to show that also freshly isolated non cultured primary and metastatic melanomas express this molecule at comparable levels, and confirm that this molecule is also expressed at low levels in certain normal *in vitro* cultured tissues, including activated macrophages and immature dendritic cells.

Early reports using binding of radiolabelled peptides (Varga *et al*, 1976; Tatro *et al*, 1990; Xia *et al*, 1996) demonstrated that the MC1R protein is present on the surface of melanoma cells. RNA coding for MC1R could also be detected in melanoma cells and in normal melanocytes (Chhajlani and Wikberg, 1992; Loir *et al*, 1999). We found that MC1R is over-expressed in the majority of the fresh melanoma tissues analysed and also in 20 out of 24 melanoma cell lines but not in carcinoma lines or LCL (Table 1). These results are in line with our earlier findings based on a smaller number of tumour lines (Salazar-Onfray *et al*, 1997). MC1R is therefore expressed in a proportion of melanomas comparable to that of the melanocytic glycoprotein gp100, a protein which has proven a promising target as a melanoma vaccine (Rosenberg *et al*, 1998). Furthermore, we found a homogeneous staining of MC1R in melanoma metastasis at various locations, indicating that this protein did not decrease in expression during tumour progression, while we were unable to detect MC1R in normal naevi, indicating that at least *in situ* melanocytes do not express detectable levels of MC1R (Figure 2A). This is in line with observations made by others, who could not detect the MC1R protein in melanocytes from normal skin by immunohistochemistry, but could easily visualize MC1R on skin with melanoma tumour growth (Xia *et al*, 1996). This demonstrates that *in vivo* melanoma cells have an increased expression of MC1R compared to normal melanocytes. The difference in levels of MC1R expression between melanoma cells and normal melanocytes could be confirmed using short time melanocyte *in vitro* cultures. The analysed melanocytes expressed detectable levels of MC1R, although the melanoma cell lines tested in parallel showed higher levels of expression (Figure 3).

The comparison between the MC1R specific mAbs with other melanoma specific mAbs commonly used in melanoma diagnosis showed that although the majority of melanomas were positive for all three mAbs (S-100, HBM45 and MC1R specific mAbs), several tumours expressed a more selective expression pattern lacking one or two of these markers. Interestingly, uveal melanoma, the most common malignant intraocular tumour in adults, can only be partially recognised by the three most widely used immunohistochemical reagents for the diagnosis (HMB-45, S-100 and A103 (Melan-A/Mart-1 specific antibody). These mAbs recognise between 25–79% of this tumour type (Nicotra *et al*, 1997; Heegaard *et al*, 2000). In comparison, our results revealed a strong expression of MC1R in all tested primary tissue sections (*n*=8) (data not shown) and uveal melanoma cell lines (Table 1). Accordingly, MC1R may constitute a valuable complementary marker for both cutaneous and uveal melanomas to be utilised in diagnosis and possibly in T cell based immunotherapy.

We have analysed a broad panel of normal tissues by immunohistochemistry and compared their expression of MC1R in relation to melanomas. Low levels of MC1R expression were detected in adrenal gland, cerebellum and liver. In addition, normal appendix, myocardium, kidney, and myometrium showed weak staining with the anti-MC1R mAb (Table 2), as did *in vitro* activated macrophages/monocytes. The immunohistochemical analysis of tissues from human skin and melanoma metastasis in brain allowed us to compare *in situ* the high expression of MC1R in melanomas with the low expression of this receptor in keratinocytes (Figure 2A). These findings are in accordance with previous findings that low levels of MC1R expression could be detected in the testis, ovary, and placenta by immunohistochemistry using MC1R specific monoclonal antibodies (Thörnvall *et al*, 1997). Also, PCR studies using specific primers have demonstrated that small amounts of the MC1R protein are synthesised by tissues other than those of the melanocytic lineage, including testis and pituitary tissues (Chhajlani, 1996) similar to what has been observed with gp100 (Brouwenstijn *et al*, 1997) and MelanA/MART1 (Busam *et al*, 1998). Taken together, these results show that this molecule is expressed in several normal tissues including *in vitro* activated macrophages of the haematopoietic system, albeit at levels substantially lower than those observed in melanomas.

In light of the relatively broad expression of MC1R in normal tissues, it is of particular interest that MC1R reactive CTL can be generated from T cells of healthy individuals (Salazar-Onfray *et al*, 1997) and melanoma patients (unpublished observation). In this context, measuring cell surface levels of MC1R is not an adequate estimation of the availability of this protein to the T cell system. Therefore, to quantify the expression of this protein by flow cytometry we have adapted an intracellular staining protocol using permeabilised cells. Melanoma lines in which the expression of these molecules by cell surface staining could not be detected by the MC1R specific mAb, including the BL line, still showed strong intracellular staining in line with previously reported radiolabelled detection by hormone homologues (Salazar-Onfray *et al*, 1997).

There is accumulating evidence that α-MSH, which is the ligand for MC1R, besides being a hormone involved in pigmentation (Halaban *et al*, 1983; Kameyama *et al*, 1989) also plays a crucial role in the regulation of immune and inflammatory reactions (Blalock, 1999; Luger *et al*, 2000). One consequence of this could be that MC1R also could be expressed on haematopoietic cells. Indeed, stimulated monocytes and macrophage lines were shown to express MC1R (Star *et al*, 1995; Bhardwaj *et al*, 1997). Our data indicated that while MC1R is not expressed at detectable levels on fresh monocytes, *in vitro* stimulation with several cytokines such as IL-4, GM-CSF, and IL-10 can induce a strong expression of this receptor (Figures 4 and 5A,B). We also confirmed the presence of MC1R in activated monocytes/macrophages and in the THP-1 cell line, but at lower levels than those found in melanomas (Figure 5A,B). Melanomas showed two times higher MC1R expression, as compared to LPS-stimulated monocytes, using a fluorescein coupled peptide hormone, and up to five times higher expression using the specific mAb MP1-1B7 in intracellular staining assays. In addition, we found it of interest that long term cultured DC derived from monocytes expressed significant levels of MC1R. One may speculate if this could reflect an activation process in which MC1R is functionally involved, since several lines of evidence indicate an important role for MC1R in inflammation (Becher *et al*, 1999; Blalock, 1999; Luger *et al*, 2000). However, although more studies are required to reveal the role of MC1R in the regulation of the immune response, this extensive analysis of MC1R tissue distribution may be of relevance not only for melanoma immunology but also dermatology, inflammation, and neuroimmunology.
